# Testing the Feasibility, Acceptability, and Potential Efficacy of an Innovative Digital Mental Health Care Delivery Model Designed to Increase Access to Care: Open Trial of the Digital Clinic

**DOI:** 10.2196/65222

**Published:** 2025-01-29

**Authors:** Natalia Macrynikola, Kelly Chen, Erlend Lane, Nic Nguyen, Jennifer Pinto, Shirley Yen, John Torous

**Affiliations:** 1 Beth Israel Deaconess Medical Center Harvard Medical School Boston, MA United States

**Keywords:** digital interventions, transdiagnostic treatment, evidence-based treatment, digital navigator, access to care, mobile phone

## Abstract

**Background:**

Mental health concerns have become increasingly prevalent; however, care remains inaccessible to many. While digital mental health interventions offer a promising solution, self-help and even coached apps have not fully addressed the challenge. There is now a growing interest in hybrid, or blended, care approaches that use apps as tools to augment, rather than to entirely guide, care. The Digital Clinic is one such model, designed to increase access to high-quality mental health services.

**Objective:**

To assess the feasibility, acceptability, and potential efficacy of the Digital Clinic model, this study aims to conduct a nonrandomized open trial with participants experiencing depression, anxiety, or both, at various levels of clinical severity.

**Methods:**

Clinicians were trained in conducting brief transdiagnostic evidence-based treatment augmented by a mental health app (mindLAMP); digital navigators were trained in supporting participants’ app engagement and digital literacy while also sharing app data with both patients and clinicians. Feasibility and acceptability of this 8-week program were assessed against a range of benchmarks. Potential efficacy was assessed by calculating pre-post change in symptoms of depression (Patient Health Questionnaire-9; PHQ-9), anxiety (7-item Generalized Anxiety Disorder; GAD-7), and comorbid depression and anxiety (Patient Health Questionnaire Anxiety and Depression Scale; PHQ-ADS), as well as rates of clinically meaningful improvement and remission. Secondary outcomes included change in functional impairment, self-efficacy in managing emotions, and flourishing.

**Results:**

Of the 258 enrolled participants, 215 (83.3%) completed the 8-week program. Most were White (n=151, 70.2%) and identified as cisgender women (n=136, 63.3%), with a mean age of 41 (SD 14) years. Feasibility and acceptability were good to excellent across a range of domains. The program demonstrated potential efficacy: the average PHQ-9 score was moderate to moderately severe at baseline (mean 13.39, SD 4.53) and decreased to subclinical (mean 7.79, SD 4.61) by the end of the intervention (*t*_126_=12.50, *P*<.001, Cohen *d*=1.11). Similarly, the average GAD-7 score decreased from moderate at baseline (mean 12.93, SD 3.67) to subclinical (mean 7.35, SD 4.19) by the end of the intervention (*t*_113_=13, *P*<.001, Cohen *d*=1.22). Participation in the program was also associated with high rates of clinically significant improvement and remission.

**Conclusions:**

Results suggest that the Digital Clinic model is feasible, acceptable, and potentially efficacious, warranting a future randomized controlled trial to establish the efficacy of this innovative model of care.

## Introduction

### Background

Common psychiatric disorders, such as depression and anxiety, are prevalent and costly. Recent nationally representative data suggest that approximately 1 in 3 adults—and 1 in 2 young adults—in the United States struggles with anxiety or depressive symptoms [1]. Although efficacious mental health treatments exist [2], demand for care outpaces its availability, leaving more than half of those with unmet mental health needs unable to access care [3]. Barriers include cost, inadequate insurance coverage, a shortage of clinicians, geographical challenges, stigma, and inadequate health care provider diversity, according to national data [4]. Alongside long-term health care system reforms, there is a need for immediate, scalable solutions to increase access to care.

Digital mental health interventions (DMHIs), delivered via smartphones or the internet, offer one promising solution. DMHIs can disseminate evidence-based interventions at scale and at low cost, and their inherent privacy can mitigate stigma. However, existing DMHIs have not yet fully realized their potential.

### Problems With Efficacy, Engagement, and Trust

DMHIs range from unguided (ie, self-help apps and electronic learning modules) to guided (those that include varying degrees of human support). As unguided DMHIs offer greater scalability at lower cost, they have been touted as ideal solutions to access problems. However, challenges with efficacy, engagement, and trust have undermined their utility in addressing access problems [5,6]. In contrast, guided DMHIs have stronger evidence of efficacy, engagement, and trust.

First, meta-analytic evidence suggests that unguided (vs guided) DMHIs have lower effect sizes and are less effective for those with more severe psychopathology [7,8]. In addition, when given a choice between using a mental health app as stand-alone treatment or to augment in-person therapy, people with moderate and severe (vs mild) psychopathology prefer the latter [9]. Even internet-delivered cognitive behavioral therapy (iCBT) with minimal human support tends to be less acceptable than individual cognitive behavioral therapy (CBT) for people with higher clinical severity [10]. These findings suggest that the lack of human support may lower perceived quality and acceptability for many, especially those with higher clinical severity.

Second, unguided DMHIs suffer from low real-world engagement. Despite demonstrating efficacy in reducing depression and anxiety in randomized controlled trials (RCTs) [11], many stand-alone mental health apps fail to sustain engagement after just 10 days in real-world settings [12]. Similarly, the dropout rates of iCBT are as high as 80%, despite iCBT’s demonstrated efficacy [13]. A recent systematic review found that DMHI engagement facilitators include greater digital literacy, more structured training in DMHI use, perceived DMHI relevance, and DMHI integration into daily life [14]. Because guided DMHIs can directly address such factors, they may be more capable of facilitating engagement [15]. Indeed, studies comparing unguided and guided versions of the same app show that the latter has better engagement and outcomes [16,17].

Third, mistrust of data handling hinders DMHI uptake [18]. Most DMHIs operate outside health care regulation and lack data use protections and transparency [18]. Examples of problematic data sharing practices and opaque privacy policies abound [19] and have, at times, led to high-profile privacy violations [20]. Such incidents undermine trust [21], contributing to low DMHI engagement [18].

Taken together, these findings suggest that sacrificing human support for scalability can lower quality and acceptability. To reach people with mental health problems that range in clinical severity, it is key to retain human support, design trustworthy DMHIs, and use scalable engagement strategies (eg, support that enhances DMHI use, relevance, and digital literacy). However, the challenge remains: how can this all be done without sacrificing scalability?

### Guided DMHIs: More Viable Solutions?

While guided DMHIs have stronger evidence, they have also been incomplete solutions for addressing barriers to access. Many simply add digital components to the existing face-to-face care [22-26], which neither lowers cost nor impacts the workforce shortage. To address these barriers, some guided DMHIs have replaced clinicians with less costly supporters (eg, coaches and nonprofessionals) [27]. While the effect sizes of those DMHIs are higher than those of unguided DMHIs [28], it remains unclear when and for whom support is best provided by a clinician, coach, or nonspecialist [29]. Until it is known, retaining a trained clinician in care remains an important ethical consideration in DMHIs.

In addition, a key implementation challenge inherent to guided DMHIs remains unresolved: As DMHIs differ from traditional in-person services, they do not readily fit within traditional clinician workflows; as such, the addition of digital tools and data can feel burdensome to clinicians [30]. As a result, when DMHIs transition from RCTs to real-world settings, uptake tends to be low, and these tools are quickly abandoned [31]. Minimizing the added workload for clinicians is essential for the successful implementation of guided DMHIs. However, examples of effective DMHI integration into health care settings remain scarce [30].

### A Practical Solution: Ensuring Scalability, Quality, Engagement, and Trust

**Figure 1:**
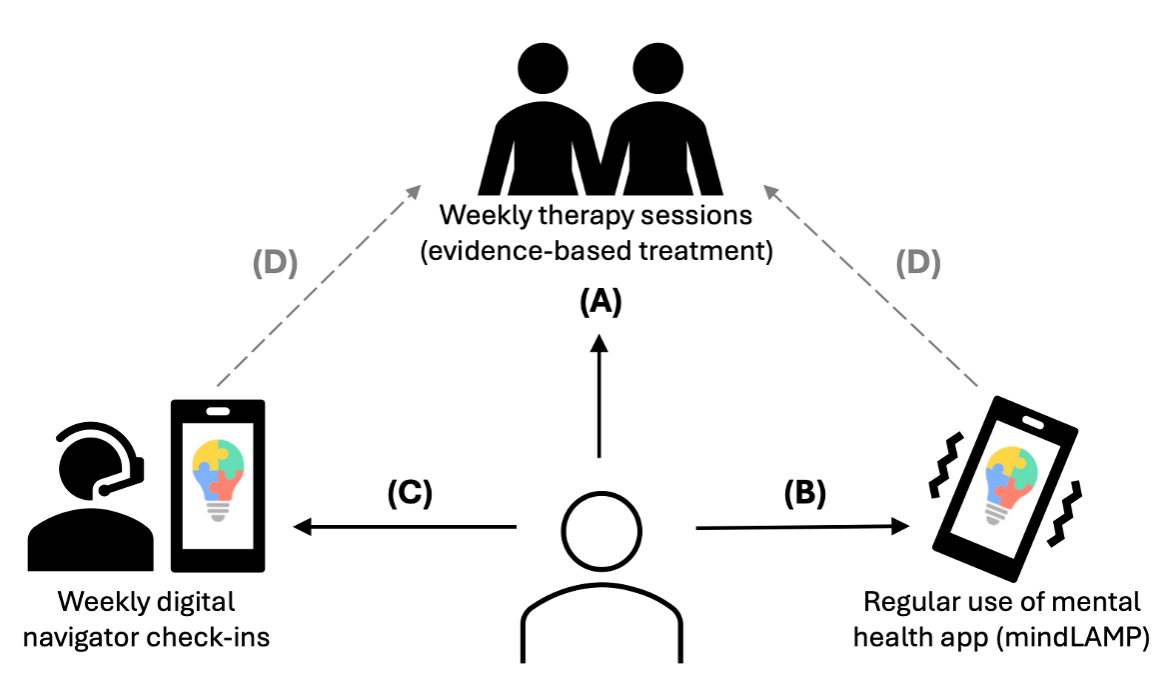
The Digital Clinic. In the Digital Clinic, (A) the patient receives brief therapy sessions of evidence-based transdiagnostic treatment, provided via telehealth by a trained clinician. (B) The mindLAMP app is integrated into care, enabling real-world skills practice and measurement-based care, including digital phenotyping data collection. (C) Brief weekly check-ins are also held via telehealth with a digital navigator, who provides technology support, shares key data insights, and encourages sustained app engagement. (D) The Digital Navigator also shares data highlights from the app with the clinician, who then uses app data to inform clinical decision-making and enhance patient care in subsequent sessions.

A more comprehensive solution is that of the Digital Clinic, an innovative guided DMHI that combines brief clinician-delivered treatment via telehealth with between-session support from an app and a nonspecialist called a digital navigator [32,33], as seen in Figure 1. Each of the model’s components addresses leading access barriers while supporting effectiveness.

First, to ensure both scalability and quality, the model offers brief evidence-based transdiagnostic treatment by a trained clinician (Figure 1A). Brief evidence-based interventions are more efficacious than treatment as usual for depression and anxiety [34-36]. Their brevity addresses the workforce shortage by freeing up clinicians faster; retaining the clinician in care increases the likelihood of reaching people with higher clinical severity.

Second, for greater scalability and impact, the mental health app mindLAMP is integrated into care to facilitate real-world skills practice and enable measurement-based care (Figure 1B). Recent meta-analytic evidence suggests that supplementing standard interventions with an app has additive effects [7], possibly increasing therapeutic dose without taking up additional clinical resources [23]. In addition, measurement-based care is a well-established yet underutilized approach to increasing care quality [37-39]. To facilitate measurement-based care, mindLAMP streamlines questionnaire administration and data visualization; data are discussed in sessions, informing clinical decision-making. The app’s digital phenotyping capacity also enables the collection of behavioral data, offering additional insights for clinical care [33].

Third, to support patient app engagement and clinician data integration into care, a digital navigator is included in this model (Figure 1C). Designed to support innovative technology-enhanced care models, the broad purpose of the digital navigator role is to make technology usable and useful for patients and clinicians [40-42]. In the Digital Clinic, digital navigators support patients during weekly check-ins in several ways: they explain data use and privacy policies to facilitate trust in mindLAMP (which is already designed with the highest privacy and security standards). They also provide technical support and app use training, as needed, share data highlights with patients, and encourage them to use the app in ways their clinician has recommended. Digital navigators also offer data insights to clinicians that can inform patient care so that data enhances care without adding to clinician workloads [43]. As digital navigators do not need clinical expertise, the addition of this feature preserves the model’s scalability and cost-effectiveness.

Finally, remote delivery methods are prioritized to enhance scalability and mitigate stigma, with therapy sessions and digital navigator check-ins conducted via telehealth. Telehealth is acceptable to patients [44], and brief evidence-based treatment via telehealth is noninferior to its in-person equivalent [45]. In addition, some data suggests that digital approaches can address stigma [46].

Prior research has not focused on or evaluated the feasibility and acceptability of a comprehensive care model incorporating all of these components to address leading access barriers. An earlier report on the development of the Digital Clinic showed that this model is promising [32]. In this study, we conducted an open trial to evaluate the Digital Clinic model’s feasibility, acceptability, and potential efficacy in treating patients with common mental health problems ranging in clinical severity.

## Methods

### Participants

A total of 10 clinicians were involved in this study (5 master’s and 3 doctoral mental health counseling students from local colleges, a postdoctoral-level clinical psychologist, and a licensed psychiatrist). Clinicians identified as White (n=6), Asian (n=3), and Black (n=1), and had a background in evidence-based treatment. In addition, 16 nonclinician volunteers with an interest in digital interventions were involved as digital navigators in the study [41,43]. Digital navigators were either college students (n=8) or had recently earned bachelor’s degrees (n=8). They had no prior clinical experience, as the digital navigator role does not require clinical expertise but rather active listening skills, which can be taught in a curriculum [42].

### Procedures

#### Recruitment and Enrollment Procedures

Individuals were screened on the basis of the inclusion and exclusion criteria shown in Textbox 1.

**Participant inclusion and exclusion criteria.**

**Inclusion criteria**

- Aged at least 18 years
- Able to speak English
- Ownership of an Android or Apple phone

**Exclusion criteria**

- Severe intellectual or attentional deficits that would interfere with participation in therapy
- Acute suicidality requiring a higher level of care
- Current enrollment in a more intensive care program (ie, inpatient treatment, partial hospitalization, or detox rehabilitation program)

For potential participants who were in higher levels of care, we recommended transitioning to the Digital Clinic after discharge, allowing it to serve as a step-down, transitional care option. Participants with subclinical anxiety or depression severity scores were not screened out to avoid denying care to those whose distress may not have been captured by these measures, but they were later excluded from the analyses.

Participants were recruited primarily through referrals from primary care at 2 hospitals in the eastern region of Massachusetts, Beth Israel Deaconess Medical Center and Beth Israel Deaconess Hospital—Needham. A psychiatrist and member of the research team held informational sessions twice a year for primary care doctors from these 2 hospitals. These sessions focused on describing the Digital Clinic, its offering, eligibility criteria, and how to introduce it to potentially eligible patients. Referrals were then made directly from primary care providers (PCPs) to the psychiatrist on the research team, with no direct outreach or recruitment of primary care patients. Recruitment began in August 2022. Upon referral receipt, participants were sent a web-based screening questionnaire via email. Once this form was completed and potential eligibility was confirmed, participants were invited to attend a virtual appointment with a trained digital navigator, who would offer details about the intervention and all of its components. If a participant chose to enroll in the clinic during this appointment, the digital navigator scheduled their first therapy appointment and administered a baseline questionnaire.

#### Intervention Procedures

Enrolled participants were offered 8 weeks of brief app-augmented treatment based on the Unified Protocol (UP) for Transdiagnostic Treatment of Emotional Disorders [47], as further described in the Intervention section below. Treatment was provided free of charge and involved weekly therapy sessions with a trained clinician, weekly check-ins with a digital navigator, and regular use of the mental health app mindLAMP. All sessions and check-ins were conducted via telehealth.

### Training and Competency

A brief therapy manual based on the UP was created for this study. This manual contained session-by-session guides, including guidance for clinicians to integrate the app and its data into care. Clinicians were first given several in-person training sessions in evidence-based treatment. These sessions, led by a licensed clinical psychologist, amounted to approximately 8 hours of training. The focus of the training was on understanding UP principles and on conducting therapy based on the manual. Once clinicians began seeing participants, their adherence and competency was monitored closely in weekly supervisions. Individual supervision (with a psychologist or psychiatrist) focused on conceptualizing patient problems within the UP and conducting treatment in line with evidence-based principles.

Digital navigators were offered 4 training sessions in person that amounted to a total of 10 hours of training. The lead digital navigator of the research team led these training sessions, which included didactic information and experiential learning through role plays. Topics covered included how to conduct the introductory session and the weekly check-ins with participants, how to troubleshoot technical issues that may arise along the way, how to understand digital phenotyping data collected via mindLAMP, and how to support digital literacy by helping participants interpret and understand their own data. Digital navigators were also trained to handle special considerations that could arise in their support meetings with clients: if a participant expressed suicidal thoughts or intent, for example, the digital navigator was trained to escalate the concern to a designated clinical supervisor. Similarly, if a client sought therapeutic advice, digital navigators were trained to encourage the participant to direct those inquiries to their clinician in the next session and to use the app for support between sessions. Upon the completion of this 10-hour training curriculum, digital navigators completed 2 supervised live appointments with participants before being approved to conduct appointments on their own. These training guidelines have been described and are published elsewhere [42,43].

### Intervention Description

The Digital Clinic is a blended care program that offers brief evidence-based treatment augmented by a mobile app (mindLAMP) [48] and a digital navigator. The purpose of integrating mindLAMP into care is 2-fold: to help patients acquire and generalize new skills and to collect psychosocial insights that inform treatment. The role of the digital navigator is to support app use, helping to resolve any difficulties (technological or motivational) that may interfere with the patient’s ability to benefit from app use. The digital navigator role has been integrated into this model in light of research showing that app engagement often correlates with clinical improvement yet tends to decline when patients are given a stand-alone app without support [18]. The digital navigator was also introduced into the model to avoid overburdening clinicians with app and data management tasks in addition to their therapy-related responsibilities.

The Digital Clinic intervention has 2 phases. Phase 1 involved app use with digital navigator support. Participants begin the 8-week program with 2 weeks of app use supported by brief weekly check-ins with a digital navigator. The goal of this period is to help participants become accustomed to completing daily and weekly self-report measures on the app, and for the app to begin collecting digital phenotyping data. Digital phenotyping data is defined as the “individual-level human phenotype in-situ using data from smartphones and other personal digital devices” [49] and in this study included several behavioral metrics: steps, movement, screentime, and a sleep estimate. Highlights of this and other data from the app were shared with participants during the digital navigator check-ins, where digital navigators also offered support for continued app use. Phase 2 involved therapy sessions and continued app use along with digital navigator support. In this phase, participants met weekly for 6 weeks with a clinician, who provided transdiagnostic treatment based on the UP. Sessions lasted between 45 and 50 minutes, with an hour-long intake. In each session, mindLAMP data were reviewed with the participant, and UP skills were discussed and assigned as home practice through the app. Therapists shared their screen at key points in each session to review mindLAMP data together with the participant, including week-by-week symptom fluctuation graphs and home practice data.

The UP was selected as the basis of care in the Digital Clinic because its transdiagnostic approach aligned with the clinic’s primary aim of increasing access to care. This approach enhances scalability by training clinicians to deliver a single therapy that can be applied to a wide range of presentations. The UP is an emotion-focused CBT that targets reactivity and avoidance, 2 underlying mechanisms that perpetuate distress across various forms of psychopathology. The therapy begins with conceptualization of the patient’s problems within the UP framework. The therapist then offers emotion psychoeducation on the adaptive function of emotions and helps the patient learn to self-monitor their mood and identify the 3 components of emotional experiences (ie, cognitive, physiological, and behavioral). The goal is to help the patient begin to tolerate and understand, rather than habitually react to, unpleasant emotions. Core UP interventions include mindfulness, cognitive flexibility, countering avoidance, and exposure (including interoceptive, emotional, and situational). A termination session consolidates learning and assists patients in creating a plan to independently practice skills and thus continue making gains after termination. The brief treatment manual designed for the Digital Clinic offers guidance on all of these topics, as seen in Table 1.

**Table 1:** Topics covered in the Digital Clinic manual^a^.

Session	Focus	mindLAMP home practice module
Intake	Problem assessment, history, goals Collaborative approach to case conceptualization based on the Unified Protocol Psychoeducation on link between 3 parts of emotional experiences (emotions or sensations, thoughts, behaviors)	Self-monitoring emotional experiences
Mindfulness	Psychoeducation on function of emotions Role of aversive reactions in maintaining emotional disorders Mindfulness practice	Mindfulness audio Anchoring in the present
Cognitive flexibility	Impact of thoughts on emotions and behavior Common thinking traps Examining automatic thoughts Practice thinking more flexibly	Thinking more flexibly
Countering avoidance	Link between emotions and behavior Avoidance and emotion-driven behaviors Opposite action practice	Act opposite
Exposure	Rationale for exposure Conduct exposure Discussion of exposures as home practice	Exposure
Termination	Progress review Plan generation for continued skills practice to maintain gains	—^b^

^a^The Digital Clinic manual focuses on emotion-focused cognitive behavioral therapy–based skill-building interventions that support adaptive coping. The manual is based on the Unified Protocol. Therapists are trained to adhere to its core principles but deliver it flexibly—that is, by slowing down the pace, emphasizing certain interventions more than others or adding an adjunctive module to tailor treatment to the patient’s needs.

^b^Not applicable, given that it is the last session, where participants were encouraged to continue practicing all skills learned during their time in the Digital Clinic.

### Materials

#### mindLAMP App and Dashboard

mindLAMP is an open-source mental health app that is designed to be easily customizable to meet the needs of different populations and to be integrated into care. mindLAMP comes with an accompanying dashboard that can be accessed on desktop by both patient and clinician. LAMP stands for the app’s 4 prominent navigation tabs: Learn (contains psychoeducation modules), Assess (self-report measures), Manage (interactive modules for skills practice), and Portal (visualizations of patient data). mindLAMP also has digital phenotyping capabilities and can automatically collect various types of behavioral data (eg, steps and screentime) without the patient having to enter it. mindLAMP has a wide variety of sensors available, including access to metrics derived from Apple Sensorkit that patients can opt in to share data from. The sensors used in the Digital Clinic are accelerometer, ambient light, nearby devices (detected through proximity to Bluetooth), and screen state. mindLAMP has been described in more detail elsewhere [32,43].

#### Digital Clinic Manuals

Clinicians were trained with the Digital Clinic manual for conducting brief, app-augmented UP-based therapy via telehealth. The manual was written by a licensed clinical psychologist and includes guidance for integrating the app and its data into care. Digital navigators were trained with the Digital Navigator manual, which details the protocol for the digital navigator role. Protocols for digital navigator sessions were previously published as part of the Digital Clinic Implementation Manual [41], along with detailed descriptions of how the digital navigator role can be adapted for different clinical settings [42].

### Measures

#### Feasibility

Feasibility of recruitment was assessed in 2 ways. First, we calculated the proportion of approached participants who enrolled in the clinic. Approached participants were those we first confirmed to be eligible upon referral and thus invited to an introductory informed consent meeting. Second, we calculated the proportion of participants who agreed to participate after understanding what is involved in the Digital Clinic program during the introductory meeting. For both metrics, a feasibility rate of at least 70% was considered good, with at least 36% considered acceptable for the first metric, given that 36% is the rate of treatment initiation upon receiving a new depressive episode diagnosis in primary care, according to large-scale research [50].

Feasibility of retention was determined by calculating the proportion of participants who completed the entire 8-week program. A feasibility rate of 70% was considered good, based on large-scale research that found a 30% attrition rate for in-person therapy in high-income countries [51]; a feasibility rate of 76% was considered ideal, based on recent RCT findings showing a 24% attrition rate for blended CBT [52] (ie, CBT that blended in-person treatment and iCBT components).

Adherence to mindLAMP home practice was determined by the frequency of activities completed in mindLAMP. Adherence was deemed good if at least 70% of participants used the app on at least half the days of their total time in the clinic (ie, 8 weeks) to complete home practice (ie, self-monitoring and UP-based skills practice). Therapist adherence to the manual was closely monitored in ongoing supervision and was deemed good if at least 75% of a random selection of 40% of all clinical notes described session content that was in line with the Digital Clinic therapy manual (ie, in line with UP core principles and interventions). Digital navigator adherence to the digital navigator protocol was assessed via checklists that each digital navigator submitted after each digital navigator meeting with a patient. Preexisting templated checklists for each check-in covered such topics as introducing the clinic structure, setting up and demonstrating the app, reviewing data highlights, and troubleshooting technology issues. Adherence was considered good if at least 75% of a random selection of 10% of all-digital navigator meeting checklists completed showed perfect adherence.

Feasibility of quantitative measures was deemed acceptable if 80% completed questionnaires at each time point. Finally, feasibility of the digital format of the program was assessed with 1 question in the postintervention questionnaire regarding hurdles to digital access (“What was the biggest hurdle you encountered regarding access to the Digital Clinic?”). Feasibility was deemed good if at least 75% endorsed “No significant hurdles encountered” rather than the other options (ie, “difficulty getting stable Wi-Fi,” “difficulty finding a quiet place for clinician sessions,” “difficulty using mindLAMP,” or other self-reported hurdles).

#### Acceptability

Satisfaction with key aspects of the intervention was evaluated using several questions in the postintervention questionnaire. For *clinician satisfaction*, participants were asked to rate “How supported did you feel by your clinician?” on a scale of 1 (not supported at all) to 5 (very supported). *Digital navigator satisfaction* was assessed with 4 items (“What was the quality of time you spent with your Digital Navigator?” “What was the quality of information provided by the Digital Navigator?” “The Digital Navigator was willing to understand my questions and concerns,” and “The Digital Navigator explained things in a way I understood.”) on a scale of 1 to 5, with 1 indicating low satisfaction and 5 high. These 4 items were averaged to create a composite digital navigator satisfaction score. *App satisfaction* was assessed with “How would you rank the mindLAMP user experience?” rated from 1 (very difficult to use) to 5 (very easy to use). Acceptability was deemed good if these components of the Digital Clinic were rated at least a 4, on average.

A total of 2 additional indicators of acceptability were assessed: *therapeutic alliance* with the clinician (measured via the Working Alliance Inventory-Short Revised; WAI-SR [53] and *digital working alliance*, or the perception of the app as a helpful therapeutic tool (Digital Working Alliance Inventory; DWAI] [54]. Both measures were administered weekly via mindLAMP, and the scores closest to the midpoint (ie, +10 or –10 days) were used in this study. For the WAI-SR, which has demonstrated good validity and reliability [55,56], participants rated 12 items (eg, “What I am doing in therapy gives me a new way to look at my problem”) from 1 (seldom) to 5 (always), summed to yield a total score ranging from 12 to 60. For DWAI, which follows the same structure as the WAI and has also shown good reliability and validity [57], participants rated 6 items (eg, “I believe the app tasks will help me to address my problem”) from 1 (strongly disagree) to 7 (strongly agree), yielding a summed total score. Although normative data for determining cutoffs for these scales are not available, it has been suggested that a score of at least a 42 is considered positive or high on the WAI-SR [58]. Our benchmarks for good acceptability thus became a minimum score of 42 on the WAI-SR and a corresponding minimum score of 30 on the DWAI, on average.

#### Potential Efficacy

Depressive symptom severity and anxiety symptom severity were assessed with the 9-item Patient Health Questionnaire (PHQ-9) [59] and the 7-item Generalized Anxiety Disorder (GAD-7) scale [60], respectively. Participants completed these measures at the pre-post and 3-month follow-up time points via a web-based questionnaire and weekly via mindLAMP during the intervention period. On each measure, participants rated from 0 (not at all) to 3 (nearly every day) how much each symptom bothered them over the past 2 weeks. Scale items were then summed to yield a total PHQ-9 score (0-27) and GAD-7 score (0-21). The PHQ-9 has demonstrated construct and criterion validity, and excellent internal reliability (α=.89) [59], as has the GAD-7 (α=.92) [60]. Scores on these 2 scales were also summed together to derive the Patient Health Questionnaire Anxiety and Depression Scale (PHQ-ADS), a measure of comorbid depressive and anxiety symptom severity with strong convergent and construct validity and high internal consistency reliability (α=.88) [61]. This measure was included as our treatment is transdiagnostic and targets comorbid disorders.

#### Secondary Clinical Outcomes

Emotion regulation self-efficacy, the hypothesized mechanism of treatment [62], was measured via the Patient Reported Outcomes Measurement Information System (PROMIS) Item Bank (version 1.0)—Self-Efficacy for Managing Emotions Short Form 8a [63], which contains 8 items rated from 1 (I am not at all confident) to 5 (I am very confident). These items are summed to yield a total score from 8 to 40, with higher scores indicating higher levels of self-efficacy for managing negative emotions. This brief scale has good psychometric properties, including high internal consistency (α=.90-.95) [63]. *Flourishing*, a measure of psychosocial functioning, was measured with the 8-item Flourishing Scale [64]. Rated from 1 (strongly disagree) to 7 (strongly agree), items on this scale are summed to yield a total score from 8 to 56, with higher scores representing greater psychological resources [65]. This scale also has good psychometric properties and high internal consistency (α=.86). *Functional impairment* was measured with the Sheehan Disability Scale, a 5-item assessment of impairment in 3 domains: work or school, social life, and family life. A total of 3 items assessing these 3 domains are rated from 0 (not at all) to 10 (extremely) and yield a total summed score of 0 (unimpaired) to 30 (highly impaired). The Sheehan Disability Scale is a psychometrically sound instrument, with good internal consistency (α=.83) [66].

### Data Analytic Plan

Analyses were conducted using R (version 4.2.1). In line with, and guided by, guidelines for feasibility studies [67], we computed descriptive statistics to assess feasibility and acceptability, and then conducted paired samples *t* tests (2-tailed, with Cohen *d* effect sizes) to examine within-group pre-post differences as a marker of potential efficacy. As commonly reported in the therapy outcomes literature, we also computed clinically significant improvement and remission rates to further assess potential efficacy. Clinically significant improvement was determined by the proportion meeting the minimum clinically important difference (MCID) thresholds established in prior empirical research, which is 4 points for the PHQ-9 and GAD-7 [61] and 6 points for the PHQ-ADS [61]. Per published guidelines, remission was defined as <8 on PHQ-9 [68] and <8 on GAD-7 [69,70], which corresponds to <16 on the PHQ-ADS.

In preparation for conducting the analyses, for participants who did not complete the questionnaire at the end of the intervention (n=36), we obtained PHQ-9 and GAD-7 scores from mindLAMP if they had completed these measures in the app within 10 days of their last therapy session (n=27). Before conducting each type of potential efficacy analysis, we excluded data from participants with baseline subclinical scores on the PHQ-9 (n=74), on the GAD-7 (n=89), or on the PHQ-ADS (n=81). A total of 7 participants experienced significant life events during the 8-week period, including the death of a close loved one (n=5, 71%) and homelessness due to eviction (n=1, 14%) or fire (n=1, 14%). Although we offered these individuals care, nonetheless, we also excluded their data from analyses as these events would have prevented adequate participation in and response to brief treatment. We also excluded data from 1 individual who was wrongly referred to the clinic for a physical rather than a psychological condition.

### Ethical Considerations

This study was reviewed before being conducted by the Beth Israel Deaconess Medical Center institutional review board and was approved as a quality improvement project (reference #2022D000016). All participants provided consent as part of the introductory meeting to the Digital Clinic, where a digital navigator informed them on all aspects of the clinic, including the collection of data and its use in the clinic and afterward. As part of the baseline questionnaire, participants signed an informed consent and acknowledgment of services form, which outlined the use of data and limits of confidentiality in treatment and asked participants for their explicit consent to have their deidentified data be used in aggregate with others’ data for research purposes. Data were collected in Health Insurance Portability and Accountability Act (HIPAA)–compliant systems. Participants did not receive compensation for participating in the Digital Clinic.

## Results

### Participants

The total number of participants who completed the Digital Clinic program was 215 (n=136, 63.3% cisgender women, n=73, 34% cisgender men, and n=6, 2.8% nonbinary), with a mean age of 41 (SD 14) years. Approximately 70.2% (151/215) identified as White, 10.7% (23/215) Asian, 9.3% (20/215) Black or African American, 7% (15/215) Hispanic or Latinx, 2.8% (6/215) Middle Eastern or North African, 0.5% (1/215) Native Hawaiian or Pacific Islander, and 0.5% (1/215) biracial.

### Feasibility of Recruitment

**Figure 2:**
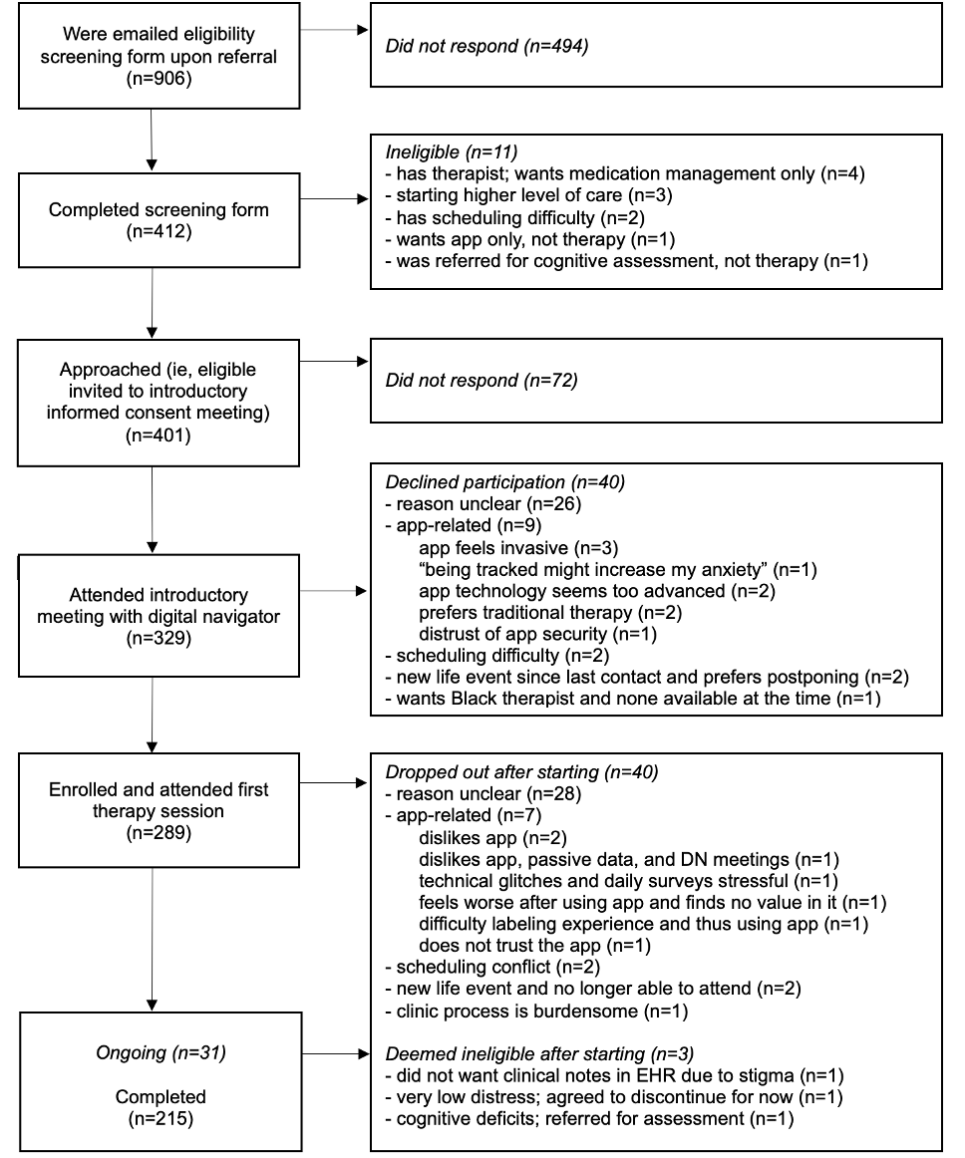
Recruitment and enrollment flowchart. Data for this study comes from a clinic with a constant flow of patients. “Ongoing” in this flowchart thus refers to participants who were still receiving treatment at the Digital Clinic at the time that data collection ended for this particular study. “Completed” refers to participants who completed a full course of care (ie, phase 1 and phase 2, as described in the Intervention Description section of the Methods section). DN: digital navigator; EHR: electronic health record.

Of the 401 individuals approached after initial eligibility was confirmed, 289 decided to enroll in the clinic—a 72.1% (289/401) recruitment rate (good, per the 70% benchmark). The proportion of participants who agreed to enroll after understanding all of the components of the Digital Clinic during their first introductory meeting was 87.8% (289/329; excellent, per the 70% benchmark). The recruitment and enrollment flowchart is shown in Figure 2.

### Feasibility of Retention

After excluding 31 participants still in progress from the 289 enrolled, 83.3% (215/258) completed the 8-week program, an excellent retention rate (given benchmarks of 70%-76%).

#### Adherence to mindLAMP Home Practice

Results indicated that 73.5% (158/215) of participants used the app on at least half the days of their total time in the clinic (benchmark 70%).

#### Therapist Adherence

A review of clinical notes from a random selection of 51 cases revealed that 86.6% (265/306) of all session notes closely adhered to UP core principles and interventions described in the Digital Clinic manual. The average adherence rate within each participant’s course of care was 87% (SD 17%), indicating that adherence was present in at least 5 of the 6 sessions, on average. Regarding the 4 core UP interventions, 98% (50/51) of cases focused on mindfulness practice, 78% (40/51) on cognitive flexibility, 76% (39/51) on countering avoidance, and 41% (21/51) on exposure. The Digital Clinic manual permits the use of non-UP adjunctive evidence-based interventions as needed, provided they align with the case conceptualization; session notes describing adjunctive interventions most often focused on assertive communication skills practice (ie, the DEARMAN [Describe, Express, Assert, Reinforce, Mindful, Appear Confident, and Negotiate] skill from dialectical behavior therapy), additional assessment and problem solving, relaxation (eg, progressive muscle relaxation and deep breathing), gratitude practice, and values clarification.

#### Digital Navigator Adherence

A review of a random selection of 22 cases’ digital navigator meeting checklists showed that 98.9% (196/198; benchmark 75%) had perfect adherence.

#### Feasibility of Quantitative Measures

For the 215 who completed the intervention, questionnaire completion rates were 100% (215/215) at baseline, 83.3% (179/215) at the end of the intervention, and 39.1% (84/215) at 3-month follow-up. (After excluding those with baseline subclinical scores on all three symptom measures [PHQ-9, GAD-7, and PHQ-ADS; n=52], the 3-month follow-up rate was still low; 68/163, 41.7%). While the baseline and postintervention questionnaire feasibility rates met and exceeded our 80% benchmark, the follow-up rate did not.

#### Feasibility of the Digital Format

Regarding hurdles to digital access, 72.1% (129/179) endorsed no significant hurdles, falling slightly below the 75% benchmark. Regarding challenges, 11.2% (20/179) reported difficulty using mindLAMP, 10.6% (19/179) difficulty finding a quiet place for clinician sessions, 3.4% (6/179) difficulty getting stable Wi-Fi, 2.2% (4/179) challenges remembering to do home practice on the app, and 0.6% (1/179) other self-reported challenges.

#### Acceptability

The average clinician, digital navigator, and mindLAMP user experience satisfaction rates were 4.81 (SD 0.52), 4.61 (SD 0.62), and 4.18 (SD 0.79), respectively (benchmark: at least a 4). The average midpoint WAI-SR and DWAI scores were 50.15 (SD 7.86) and 32.10 (SD 6.74), respectively, exceeding the benchmarks of 42 (WAI-SR) and 30 (DWAI).

#### Potential Efficacy

For those who entered the clinic with depression severity in the clinical range, the average baseline PHQ-9 score was in the moderate to moderately severe range (mean 13.39, SD 4.53) and fell to the subclinical range (mean 7.79, SD 4.61) by the end of the intervention, a statistically significant mean reduction of 5.61 (95% CI 4.72-6.49; *t*_126_=12.50; *P*<.001), with a large effect size (Cohen *d*=1.11). Gains were maintained at the 3-month follow-up for those who completed the follow-up questionnaire and had an end-of-intervention PHQ-9 score (n=55, 43%), with scores still in the subclinical range (mean 7.42, SD 4.60) and not significantly different from scores at the end of the intervention (*t*_54_=1.38; *P*=.17).

For those who entered the clinic with anxiety severity in the clinical range, the average baseline GAD-7 score was in the moderate range (mean 12.93, SD 3.67) and fell to the subclinical range (mean 7.35, SD 4.19) by the end of the intervention, a statistically significant mean reduction of 5.58 (95% CI 4.73-6.43; *t*_113_=13; *P*<.001), with a large effect size (Cohen *d*=1.22). Gains were maintained at 3-month follow-up for those who completed the follow-up questionnaire and had a postintervention GAD-7 score (n=48, 42%), with scores still in the subclinical range (mean 6.88, SD 4.65) and not significantly different from scores at the end of the intervention (*t*_47_=0.62; *P*=.54).

For those who entered the clinic with comorbid depressive and anxiety symptoms in the clinical range, the average baseline PHQ-ADS score was in the moderate range (mean 25.51, SD 7.05) and fell to the subclinical range (mean 15.01, SD 8.30) by the end of the intervention, a statistically significant mean reduction of 10.50 (95% CI 8.95-12; *t*_121_=13.40; *P*<.001), with a large effect size (Cohen *d*=1.21). Gains were maintained at 3-month follow-up for those who completed the follow-up questionnaire and had a postintervention PHQ-ADS score (n=52; 43%), with scores still in the subclinical range at 3-month follow-up (M 14.56, SD 8.77) and not significantly different from scores at the end of the intervention (*t*_51_=1.09; *P*=.28).

From baseline to the end of the intervention, approximately 68.5% (87/127), 76.3% (87/114), and 70.5% (86/122) of those who completed the intervention experienced at least a 25% symptom decrease in PHQ-9, GAD-7, and PHQ-ADS, respectively. Rates of clinically significant improvement (ie, a reduction equal to or greater than the MCID) were 63.8% (81/127), 66.7% (76/114), and 73% (89/122) in terms of PHQ-9, GAD-7, and PHQ-ADS, respectively. Remission rates were 52.8% (67/127), 59.6% (68/114), and 55.7% (68/122) on the PHQ-9, GAD-7, and PHQ-ADS, respectively. These per protocol rates along with the intent-to-treat rates are shown in Table 2 and the rates calculated separately by baseline severity level are shown in Table 3.

**Table 2:** Rates of overall clinically significant improvement and remission (N=215).

	Values, n (%)	T1^a^, mean (SD)	T2^b^, mean (SD)	Clinically significant change^c^ (%)	Remission^d^ (%)	Remission or mild at T2^e^ (%)
Dep^f^ (PP^g^; n=134)	127 (95)	13.39 (4.53)	7.79 (4.61)	64	53	66
Anx^h^ (PP; n=120)	114 (95)	12.93 (3.67)	7.35 (4.19)	67	60	73
Comorb^i^ (PP; n=127)	122 (96)	25.51 (7.05)	15.01 (8.30)	73	56	70
Dep (ITT^j,k^ MI^l^)	161 (100)	13.55 (4.52)	7.86 (4.16)	66	55	73
Anx (ITT MI)	148 (100)	13.25 (3.78)	7.44 (3.74)	73	59	79
Comorb (ITT MI)	155 (100)	26.08 (7.23)	15.26 (7.50)	77	56	77
Dep (ITT BOCF^m,n^)	161 (100)	13.55 (4.52)	9.11 (5.27)	50	42	53
Anx (ITT BOCF)	148 (100)	13.25 (3.78)	8.92 (5.08)	53	47	57
Comorb (ITT BOCF)	155 (100)	26.08 (7.23)	17.82 (9.78)	57	44	55

^a^T1: baseline time point.

^b^T2: end-of-intervention time point.

^c^Clinically significant improvement, also known as clinically meaningful change.

^d^Remission: rates of remission at T2.

^e^Remission or mild at T2: proportion of participants whose T2 symptoms were mild or in remission if baseline symptoms were moderate or severe, or in remission if baseline symptoms were mild.

^f^Dep: depressive symptoms, measured by the 9-item Patient Health Questionnaire scale.

^g^PP: per protocol sample (ie, participants with a baseline clinical-range score who completed the intervention and had a T2 score). Percentages represent the proportion of PP participants out of all completers with a baseline clinical-range score, regardless of T2 questionnaire completion).

^h^Anx: anxiety symptoms, measured by the 7-item Generalized Anxiety Disorder scale.

^i^Comorb: comorbid symptoms of depression of anxiety, measured by the PHQ-Anxiety and Depression Scale.

^j^ITT: intent-to-treat.

^k^ITT: intent-to-treat sample, which includes those with missing T2 scores either due to dropout or T2 questionnaire noncompletion.

^l^ITT MI: missing T2 scores were imputed using the mean imputation method (ie, the PP T2 mean of the severity subgroup to which the participant belongs).

^m^BOCF: baseline observation carried forward.

^n^ITT BOCF: missing T2 scores were imputed using the baseline observation carried forward method, a conservative worst-case scenario approach that assumes participants with missing T2 scores would have had no improvement.

**Table 3:** Potential efficacy rates (N=215).

Baseline severity		Values, n (%)	T1^a^, mean (SD)	T2^b^, mean (SD)	Clinically significant change^c^ (%)	Remission^d^ (%)	Remission or mild at T2^e^ (%)
**Depressive symptoms (PHQ-9^f^)**							
	Mild (PP^g^; n=33)	32 (97)	8.53 (0.51)	5.66 (2.82)	69	69	—^h^
	Moderate (PP; n=56)	53 (95)	11.89 (1.41)	7.34 (4.02)	62	55	72
	Severe (PP; n=44)	42 (95)	19.00 (2.60)	9.98 (5.49)	81	38	57
	Mild (ITT^i,j^ MI^k,l^)	36 (100)	8.50 (0.51)	5.66 (2.66)	72	72	—
	Moderate (ITT MI)	70 (100)	11.83 (1.35)	7.34 (3.49)	66	66	76
	Severe (ITT MI)	55 (100)	19.05 (2.54)	9.98 (4.78)	85	29	67
	Mild (ITT BOCF^m,n^)	36 (100)	8.50 (0.51)	5.88 (2.77)	64	64	—
	Moderate (ITT BOCF)	70 (100)	11.83 (1.35)	8.39 (3.99)	47	41	53
	Severe (ITT BOCF)	55 (100)	19.05 (2.54)	12.16 (6.31)	62	29	44
**Anxiety symptoms (GAD-7^o^)**							
	Mild (PP; n=23)	22 (96)	8.45 (0.51)	4.68 (2.44)	86	86	—
	Moderate (PP; n=58)	56 (97)	11.73 (1.43)	7.27 (3.75)	66	57	79
	Severe (PP; n=39)	36 (92)	17.53 (1.86)	9.11 (4.82)	81	47	56
	Mild (ITT MI)	26 (100)	8.46 (0.51)	4.68 (2.23)	88	88	—
	Moderate (ITT MI)	72 (100)	11.83 (1.39)	7.27 (3.30)	72	67	83
	Severe (ITT MI)	50 (100)	17.78 (1.96)	9.11 (4.07)	86	34	68
	Mild (ITT BOCF)	26 (100)	8.46 (0.51)	5.10 (2.54)	77	77	—
	Moderate (ITT BOCF)	72 (100)	11.83 (1.39)	8.36 (3.93)	51	44	61
	Severe (ITT BOCF)	50 (100)	17.78 (1.96)	11.72 (5.97)	58	34	40
**Comorbid depressive and anxiety symptoms (PHQ-ADS^p^)**							
	Mild (PP)	30 (100)	17.53 (1.07)	9.93 (5.57)	80	80	—
	Moderate (PP; n=58)	56 (97)	24.02 (2.90)	15.09 (6.62)	66	48	71
	Severe (PP; n=38)	36 (95)	34.87 (4.09)	19.11 (10.20)	86	47	61
	Mild (ITT MI)	34 (100)	17.56 (1.08)	9.93 (5.57)	80	80	—
	Moderate (ITT MI)	71 (100)	24.06 (2.91)	15.09 (5.87)	70	59	77
	Severe (ITT MI)	50 (100)	34.76 (4.27)	19.11 (8.62)	90	34	72
	Mild (ITT BOCF)	34 (100)	17.56 (1.08)	10.85 (5.83)	71	71	—
	Moderate (ITT BOCF)	71 (100)	24.06 (2.91)	17.01 (7.10)	52	38	56
	Severe (ITT BOCF)	50 (100)	34.76 (4.27)	23.70 (11.65)	62	34	44

^a^T1: baseline time point.

^b^T2: posttreatment time point.

^c^Clinically significant improvement, also known as clinically meaningful change.

^d^Remission: rates of remission at T2.

^e^Remission or mild at T2: proportion of participants whose T2 symptoms were either at least in the mild range (if moderate or severe at baseline) or in remission (if mild at baseline).

^f^PHQ-9: 9-item Patient Health Questionnaire.

^g^Per-protocol sample (ie, participants who completed the intervention and had a T2 score).

^h^Not applicable.

^i^ITT: intent-to-treat.

^j^Intent-to-treat sample, which includes those with missing T2 scores (either due to dropout or questionnaire noncompletion).

^k^MI: mean imputation.

^l^ITT MI: missing T2 scores were imputed using the mean imputation method (ie, the PP T2 mean of the severity subgroup to which the participant belongs).

^m^BOCF: baseline observation carried forward.

^n^ITT BOCF: missing T2 scores were imputed using the baseline observation carried forward method, a conservative worst-case scenario approach that assumes participants with missing T2 scores would have had no improvement.

^o^GAD-7: 7-item Generalized Anxiety Disorder Scale.

^p^PHQ-ADS: PHQ-Anxiety and Depression Scale.

#### Secondary Clinical Outcomes

Emotion regulation self-efficacy significantly increased from baseline (mean 20.42, SD 4.99) to the end of the intervention (mean 26.97 SD 5.61; *t*_133_=–13.13; *P*<.001). Flourishing significantly rose from baseline (mean 39.90, SD 9.06) to the end of the intervention (mean 44.43, SD 8.28; *t*_83_=–6.06; *P*<.001). Functional impairment significantly decreased from baseline (mean 18.72, SD 6.41) to the end of the intervention (mean 13.24 SD 8.30; *t*_134_=8.94; *P*<.001). Gains were maintained at 3-month follow-up (mean 9.86, SD 7.68), with scores significantly lower at the follow-up time point (*t*_59_=3.86; *P*<.001).

## Discussion

### Overview

This study was an initial evaluation of the Digital Clinic, an innovative model of care designed to mitigate leading mental health care access barriers by offering brief, clinician-delivered evidence-based treatment via telehealth that is augmented by an app and a digital navigator [34,71]. The aim of this study was to assess the feasibility, acceptability, and potential efficacy of this model by conducting a nonrandomized open trial. Results suggest that this model is feasible, acceptable, and has the potential to be an effective solution for increasing access to high-quality, evidence-based mental health intervention.

### Principal Findings and Comparison With Prior Work

The open trial showed good to excellent feasibility and acceptability across most metrics. Results indicated that it is feasible to recruit and retain patients. About 87.8% (289/329) of the participants chose to continue after the introductory meeting, which suggests that many patients were not discouraged by the clinic’s all-digital format or its various innovative components (ie, app, digital phenotyping data collection, and integration of a digital navigator into care). Moreover, the Digital Clinic’s completion rate met and exceeded rates seen in other guided DMHIs (eg, blended CBT [52]) and in-person therapy [72]. It also improved on attrition rates of unguided DMHIs, which reach nearly 50% when accounting for publication bias [73].

Therapist, digital navigator, and mindLAMP adherence were also all high. That therapists were not all highly experienced suggests that the training procedures and clinic manual effectively enabled delivery of brief, transdiagnostic, evidence-based treatment by clinicians with a range of experience, supporting the scalability of the model. That digital navigator adherence was also high suggests that checklists helped facilitate smooth check-ins and should be used in future studies. Finally, mindLAMP adherence among participants was high, with app use between sessions potentially extending the impact of treatment to their daily lives. In addition, these 3 components of the clinic (app, clinician, and digital navigator) appeared to be acceptable to patients, based on high satisfaction ratings with each one.

In terms of the potential efficacy of the Digital Clinic, the results yielded promising findings. The average depression, anxiety, and comorbid depression and anxiety symptom scores fell to the subclinical range by the end of the intervention, suggesting the potential of the DMHI in making a meaningful clinical impact in a short time. Moreover, overall rates of clinically significant improvement (67% and 64% for anxiety and depressive symptoms, respectively) and remission (60% and 53% for anxiety and depressive symptoms, respectively) were high, meeting and exceeding those typically observed in traditional treatment (ie, 51% remission for anxiety disorders [74] and 33% for depression [75], according to recent meta-analyses of primarily evidence-based treatments). However, this feasibility study was not designed to establish efficacy and thus did not use a control group. Nevertheless, these outcomes are promising and warrant a future RCT to establish the efficacy.

### Lessons Learned

While the feasibility of quantitative measures was high for baseline and end-of-intervention time points, completion rate of the 3-month follow-up measures fell below our benchmark. This suggests room for improvement, with our current method of simple email outreach 3 months after the intervention being insufficient. Alternative strategies for a future study include offering participants payment or scheduling a study appointment for the 3-month follow-up questionnaire upfront.

Further room for improvement was suggested in terms of the feasibility of aspects of the digital format. Overall, 72.1% (129/179) endorsed that they encountered no significant hurdles, slightly below our 75% benchmark. Most hurdles reported were around finding a quiet place for therapy sessions (19/179, 10.6%) and difficulty using mindLAMP (6/179, 3.4%). To mitigate the first barriers, in future studies, it will help to build in protected time at the introductory meeting to discuss and resolve practical barriers with each patient around finding privacy for therapy sessions. Regarding the second hurdle, it is likely that difficulty reported around mindLAMP use was resolved in digital navigator meetings. Nevertheless, a future study analyzing the qualitative feedback obtained in this study will help shed light on this question and inform refinements.

Finally, a key lesson about DMHI implementation came out of this study: Partnering with primary care substantially helped with the successful implementation of the Digital Clinic. For one, it eased the burden of delivering mental health care on PCPs—a task that increasingly falls on them due to limited access to specialty mental health care [76]. In addition, PCPs reported that strongly endorsing the Digital Clinic directly to their patients rather than referring them to an external resource seemed to increase patients’ willingness to start care at the Digital Clinic. These PCPs noted that having the Digital Clinic embedded into the health care system may have felt like a natural extension of patients’ primary care received at the hospital, rather than being viewed as a separate mental health program. Thus, partnering with primary care can solve provider problems and increase patient engagement and access to mental health care.

### Strengths and Limitations

Strengths of this study include its evaluation of a program designed to be both highly scalable and effective, offering a promising solution to increase access to care. Several components increase scalability, including brief therapy, the creation of standardized manuals for the clinician and digital navigator roles, and the use of an open-source smartphone app. The focus on transdiagnostic evidence-based treatment enhances the program’s ability to reach individuals with a wide range of clinical presentations. That the program was effective for a majority of this patient sample without the need for highly experienced clinicians supports the potential for its widespread adoption and implementation in various health care settings.

Despite these strengths, several limitations are also worth considering. First, although valid and reliable instruments were used for all outcomes, the PHQ-9 and GAD-7 measures do not adequately capture distress for some clinical presentations (eg, someone with social anxiety disorder, whose distress is only apparent in specific situations they may generally avoid). Future studies should consider a fuller breadth of outcome measures. Second, the low ethonoracial diversity of our sample may limit the applicability of the findings to more diverse populations. In addition, this study focused entirely on English-speaking participants, and the experiences and outcomes discussed in this study may not be fully reflective of those of non-English speakers. These factors should therefore be considered when interpreting the generalizability of this study’s findings. Third, because this was a feasibility study [77] designed to primarily examine whether this model is feasible and acceptable to patients and providers (and secondarily whether it is *potentially* efficacious), no control group was used. The lack of a control group precludes definitive conclusions about clinical improvement resulting from the intervention itself rather than the passage of time or other factors. Fourth, we did not track treatment fidelity in a traditional manner, relying instead on supervision meetings and the subsequent examination of clinical notes to assess clinician adherence. This method could be strengthened in a future study by using a checklist system as done with digital navigators, or by obtaining session recordings coded for adherence. Fourth, although data integration into care was a frequent topic of discussion in supervision meetings, we did not formally track the extent to which clinicians regularly incorporated data into care. This limitation paves the way toward fruitful avenues for further enhancing clinician training in the future and implementing a process of actively measuring the extent of data incorporation into care. Finally, we did not formally track whether patients were concurrently receiving other therapies or medications. We inquired about these factors at intake, and in the few cases in which participants were in concurrent outpatient therapy, we recommended that they start in the Digital Clinic after their current treatment. However, we still accommodated those on medications. As a result, for some patients, pre-post clinical improvements may not be solely due to the Digital Clinic intervention. This aspect will need to be more rigorously tracked and addressed in a future RCT.

### Future Directions

Results of this study suggest that this model of care shows promise as a solution for mitigating barriers to quality mental health intervention access. This model was informed by research that shows that digital interventions offered with (vs without) human support are more effective [7], especially for people with higher clinical severity [31]. The inclusion of support in terms of clinicians and digital navigators appears to be effective in increasing access and quality in care for people with a range of clinical severity. Results suggest that the Digital Clinic warrants a future efficacy RCT.

Another future direction is to conduct a dismantling study to isolate the additive effects on outcomes of the brief clinician-delivered therapy, the digital navigator, and mindLAMP app integration. Research suggests that the digital components of guided DMHIs may independently contribute to outcomes [78] and that mindLAMP enhanced by a digital navigator alone can have its own beneficial effects [79]. The extent of each component’s impact on efficacy can inform future model adaptation and lead to a more cost-effective, stepped-care version of the Digital Clinic, where more clinical resources are offered first to those with higher severity. Recent research supports such models, as they use limited clinical resources in ways that maximize efficacy and are cost-effective [80].

Related, continuing to focus on the implementation and therapeutic effect of incorporating digital phenotyping data versus actively collected data into care is an important future direction. We observed that some clinician-patient dyads embraced both types of data more readily than others, with some patients also benefiting more from digital phenotyping aspects of the data than others. We also observed that some clinicians required further training to feel more confident in integrating all kinds of data into care. This is not surprising, as most training programs do not prioritize the development of skills in conducting measurement-based care. Addressing these implementation and knowledge gaps may enhance both clinician readiness and patient outcomes.

Finally, a still unanswered question in the field is whether gains obtained from participating in brief, guided DMHIs are sustained in the long term. In this study, clinical improvement was sustained for the 41.7% (68/163) of participants with baseline clinical scores who completed the 3-month follow-up questionnaire. This finding once again suggests that this intervention may be potentially efficacious in the short and long term, but it warrants more research to confirm this possibility.

### Conclusions

Guided DMHIs that offer brief, evidence-based treatment may reduce the cost of care and alleviate the provider shortage; integrating an app into treatment that provides between-session support and enables measurement-based care can increase the impact of care delivered in a short time. The digital navigator supports app engagement and can lower the burden of data collection and technology use on the clinician. Results from this study suggest that the Digital Clinic model is feasible, acceptable, and potentially efficacious, warranting a future RCT.

## Abbreviations

CBT: cognitive behavioral therapy
DEARMAN: Describe, Express, Assert, Reinforce, Mindful, Appear Confident, and Negotiate
DMHI: digital mental health intervention
DWAI: Digital Working Alliance Inventory
GAD-7: 7-item Generalized Anxiety Disorder
HIPAA: Health Insurance Portability and Accountability Act
iCBT: internet-delivered cognitive behavioral therapy
MCID: minimum clinically important difference
PCP: primary care provider
PHQ-9: 9-item Patient Health Questionnaire
PHQ-ADS: Patient Health Questionnaire Anxiety and Depression Scale
PROMIS: Patient Reported Outcomes Measurement Information System
RCT: randomized controlled trial
UP: Unified Protocol
WAI-SR: Working Alliance Inventory-Short Revised

## Data Availability

The datasets generated and analyzed during this study are not available due to the clinical nature of this project.

## References

[1] (2023). Latest federal data show that young people are more likely than older adults to be experiencing symptoms of anxiety or depression. Kaiser Family Foundation.

[2] (2022). Psychological treatments. Society of Clinical Psychology, Division 12, American Psychological Association.

[3] (2020). Key substance use and mental health indicators in the United States: results from the 2020 National Survey on Drug Use and Health. Substance Abuse and Mental Health Services Administration (SAMHSA).

[4] Lopes L, Kirzinger A, Sparks G, Stokes M, Brodie M KFF/CNN mental health in America survey. Kaiser Family Foundation (KFF).

[5] Smith KA, Blease C, Faurholt-Jepsen M, Firth J, Van Daele T, Moreno C, Carlbring P, Ebner-Priemer UW, Koutsouleris N, Riper H, Mouchabac S, Torous J, Cipriani A (2023). Digital mental health: challenges and next steps. BMJ Ment Health.

[6] Torous J, Bucci S, Bell IH, Kessing LV, Faurholt-Jepsen M, Whelan P, Carvalho AF, Keshavan M, Linardon J, Firth J (2021). The growing field of digital psychiatry: current evidence and the future of apps, social media, chatbots, and virtual reality. World Psychiatry.

[7] Linardon J, Cuijpers P, Carlbring P, Messer M, Fuller-Tyszkiewicz M (2019). The efficacy of app-supported smartphone interventions for mental health problems: a meta-analysis of randomized controlled trials. World Psychiatry.

[8] Karyotaki E, Efthimiou O, Miguel C, Bermpohl FM, Furukawa TA, Cuijpers P, Riper H, Patel V, Mira A, Gemmil AW, Yeung AS, Lange A, Williams AD, Mackinnon A, Geraedts A, van Straten A, Meyer B, Björkelund C, Knaevelsrud C, Beevers CG, Botella C, Strunk DR, Mohr DC, Ebert DD, Kessler D, Richards D, Littlewood E, Forsell E, Feng F, Wang F, Andersson G, Hadjistavropoulos H, Christensen H, Ezawa ID, Choi I, Rosso IM, Klein JP, Shumake J, Garcia-Campayo J, Milgrom J, Smith J, Montero-Marin J, Newby JM, Bretón-López J, Schneider J, Vernmark K, Bücker L, Sheeber LB, Warmerdam L, Farrer L, Heinrich M, Huibers MJ, Kivi M, Kraepelien M, Forand NR, Pugh N, Lindefors N, Lintvedt O, Zagorscak P, Carlbring P, Phillips R, Johansson R, Kessler RC, Brabyn S, Perini S, Rauch SL, Gilbody S, Moritz S, Berger T, Pop V, Kaldo V, Spek V, Forsell Y, Individual Patient Data Meta-Analyses for Depression (IPDMA-DE) Collaboration (2021). Internet-based cognitive behavioral therapy for depression: a systematic review and individual patient data network meta-analysis. JAMA Psychiatry.

[9] Nelson BW, Peiper NC, Forman-Hoffman VL (2024). Digital mental health interventions as stand-alone vs. augmented treatment as usual. BMC Public Health.

[10] Cuijpers P, Noma H, Karyotaki E, Cipriani A, Furukawa TA (2019). Effectiveness and acceptability of cognitive behavior therapy delivery formats in adults with depression: a network meta-analysis. JAMA Psychiatry.

[11] Goldberg SB, Lam SU, Simonsson O, Torous J, Sun S (2022). Mobile phone-based interventions for mental health: a systematic meta-review of 14 meta-analyses of randomized controlled trials. PLOS Digit Health.

[12] Baumel A, Muench F, Edan S, Kane JM (2019). Objective user engagement with mental health apps: systematic search and panel-based usage analysis. J Med Internet Res.

[13] Saad A, Bruno D, Camara B, D'Agostino J, Bolea-Alamanac B (2021). Self-directed technology-based therapeutic methods for adult patients receiving mental health services: systematic review. JMIR Ment Health.

[14] Borghouts J, Eikey E, Mark G, De Leon C, Schueller SM, Schneider M, Stadnick N, Zheng K, Mukamel D, Sorkin DH (2021). Barriers to and facilitators of user engagement with digital mental health interventions: systematic review. J Med Internet Res.

[15] Gan DZ, McGillivray L, Larsen ME, Christensen H, Torok M (2022). Technology-supported strategies for promoting user engagement with digital mental health interventions: a systematic review. Digit Health.

[16] Benjet C, Zainal NH, Albor Y, Alvis-Barranco L, Carrasco-Tapias N, Contreras-Ibáñez CC, Cudris-Torres L, de la Peña FR, González N, Guerrero-López JB, Gutierrez-Garcia RA, Jiménez-Peréz AL, Medina-Mora ME, Patiño P, Cuijpers P, Gildea SM, Kazdin AE, Kennedy CJ, Luedtke A, Sampson NA, Petukhova MV, Kessler RC (2023). A precision treatment model for internet-delivered cognitive behavioral therapy for anxiety and depression among university students: a secondary analysis of a randomized clinical trial. JAMA Psychiatry.

[15] Papola D, Ostuzzi G, Tedeschi F, Gastaldon C, Purgato M, Del Giovane C, Pompoli A, Pauley D, Karyotaki E, Sijbrandij M, Furukawa Ta, Cuijpers P, Barbui C (2023). CBT treatment delivery formats for panic disorder: a systematic review and network meta-analysis of randomised controlled trials. Psychol Med.

[18] Torous J, Firth J, Goldberg SB (2024). Digital mental health's unstable dichotomy-wellness and health. JAMA Psychiatry.

[19] Huckvale K, Torous J, Larsen ME (2019). Assessment of the data sharing and privacy practices of smartphone apps for depression and smoking cessation. JAMA Netw Open.

[20] Federal Trade Commission. Alcohol addiction treatment firm will be banned from disclosing health data for advertising to settle FTC charges that it shared data without consent.

[21] Ghafur S, Van Dael J, Leis M, Darzi A, Sheikh A (2020). Public perceptions on data sharing: key insights from the UK and the USA. Lancet Digit Health.

[22] Kemmeren LL, van Schaik A, Draisma S, Kleiboer A, Riper H, Smit JH (2023). Effectiveness of blended cognitive behavioral therapy versus treatment as usual for depression in routine specialized mental healthcare: E-COMPARED trial in the Netherlands. Cogn Ther Res.

[23] Berger T, Krieger T, Sude K, Meyer B, Maercker A (2018). Evaluating an e-mental health program ("deprexis") as adjunctive treatment tool in psychotherapy for depression: results of a pragmatic randomized controlled trial. J Affect Disord.

[24] Kooistra LC, Wiersma JE, Ruwaard J, Neijenhuijs K, Lokkerbol J, van Oppen P, Smit F, Riper H (2019). Cost and effectiveness of blended versus standard cognitive behavioral therapy for outpatients with depression in routine specialized mental health care: pilot randomized controlled trial. J Med Internet Res.

[25] Witlox M, Garnefski N, Kraaij V, de Waal MW, Smit F, Bohlmeijer E, Spinhoven P (2021). Blended acceptance and commitment therapy versus face-to-face cognitive behavioral therapy for older adults with anxiety symptoms in primary care: pragmatic single-blind cluster randomized trial. J Med Internet Res.

[26] Erbe D, Eichert HC, Riper H, Ebert DD (2017). Blending face-to-face and internet-based interventions for the treatment of mental disorders in adults: systematic review. J Med Internet Res.

[27] Roos LG, Sagui-Henson SJ, Castro Sweet C, Welcome Chamberlain CE, Smith BJ (2024). Improvement and maintenance of clinical outcomes in a digital mental health platform: findings from a longitudinal observational real-world study. JMIR Mhealth Uhealth.

[28] Leung C, Pei J, Hudec K, Shams F, Munthali R, Vigo D (2022). The effects of nonclinician guidance on effectiveness and process outcomes in digital mental health interventions: systematic review and meta-analysis. J Med Internet Res.

[29] Werntz A, Amado S, Jasman M, Ervin A, Rhodes JE (2023). Providing human support for the use of digital mental health interventions: systematic meta-review. J Med Internet Res.

[30] Connolly SL, Kuhn E, Possemato K, Torous J (2021). Digital clinics and mobile technology implementation for mental health care. Curr Psychiatry Rep.

[31] Lattie EG, Stiles-Shields C, Graham AK (2022). An overview of and recommendations for more accessible digital mental health services. Nat Rev Psychol.

[32] Macrynikola N, Nguyen N, Lane E, Yen S, Torous J (2023). The digital clinic: an innovative mental health care delivery model utilizing hybrid synchronous and asynchronous treatment. NEJM Catal.

[33] Torous J, Hsin H (2018). Empowering the digital therapeutic relationship: virtual clinics for digital health interventions. NPJ Digit Med.

[34] Bisby MA, Balakumar T, Scott AJ, Titov N, Dear BF (2024). An online therapist-guided ultra-brief treatment for depression and anxiety: a randomized controlled trial. Psychol Med.

[35] Cully JA, Stanley MA, Petersen NJ, Hundt NE, Kauth MR, Naik AD, Sorocco K, Sansgiry S, Zeno D, Kunik ME (2017). Delivery of brief cognitive behavioral therapy for medically ill patients in primary care: a pragmatic randomized clinical trial. J Gen Intern Med.

[36] Nieuwsma JA, Trivedi RB, McDuffie J, Kronish I, Benjamin D, Williams JW (2012). Brief psychotherapy for depression: a systematic review and meta-analysis. Int J Psychiatry Med.

[37] Lewis CC, Boyd M, Puspitasari A, Navarro E, Howard J, Kassab H, Hoffman M, Scott K, Lyon A, Douglas S, Simon G, Kroenke K (2019). Implementing measurement-based care in behavioral health: a review. JAMA Psychiatry.

[38] Torous J, Powell AC, Rodriguez-Villa E (2020). Health information technology resources to support measurement-based care. Child Adolesc Psychiatr Clin N Am.

[39] Shimokawa K, Lambert MJ, Smart DW (2010). Enhancing treatment outcome of patients at risk of treatment failure: meta-analytic and mega-analytic review of a psychotherapy quality assurance system. J Consult Clin Psychol.

[40] Rodriguez JA, Charles JP, Bates DW, Lyles C, Southworth B, Samal L (2023). Digital healthcare equity in primary care: implementing an integrated digital health navigator. J Am Med Inform Assoc.

[41] Perret S, Alon N, Carpenter-Song E, Myrick K, Thompson K, Li S, Sharma K, Torous J (2023). Standardising the role of a digital navigator in behavioural health: a systematic review. Lancet Digit Health.

[42] Wisniewski H, Gorrindo T, Rauseo-Ricupero N, Hilty D, Torous J (2020). The role of digital navigators in promoting clinical care and technology integration into practice. Digit Biomark.

[43] Chen K, Lane E, Burns J, Macrynikola N, Chang S, Torous J (2024). The digital navigator: standardizing human technology support in app-integrated clinical care. Telemed J E Health.

[44] Polinski JM, Barker T, Gagliano N, Sussman A, Brennan TA, Shrank WH (2016). Patients' satisfaction with and preference for telehealth visits. J Gen Intern Med.

[45] Swartz HA, Bylsma LM, Fournier JC, Girard JM, Spotts C, Cohn JF, Morency L (2023). Randomized trial of brief interpersonal psychotherapy and cognitive behavioral therapy for depression delivered both in-person and by telehealth. J Affect Disord.

[46] Kim HM, Xu Y, Wang Y (2022). Overcoming the mental health stigma through m-Health apps: results from the healthy minds study. Telemed J E Health.

[47] Barlow DH, Harris BA, Eustis EH, Farchione TJ (2020). The unified protocol for transdiagnostic treatment of emotional disorders. World Psychiatry.

[48] Vaidyam A, Halamka J, Torous J (2022). Enabling research and clinical use of patient-generated health data (the mindLAMP platform): digital phenotyping study. JMIR Mhealth Uhealth.

[49] Torous John, Kiang Mathew V, Lorme Jeanette, Onnela Jukka-Pekka (2016). New Tools for New Research in Psychiatry: A Scalable and Customizable Platform to Empower Data Driven Smartphone Research. JMIR Ment Health.

[50] Waitzfelder B, Stewart C, Coleman KJ, Rossom R, Ahmedani BK, Beck A, Zeber JE, Daida YG, Trinacty C, Hubley S, Simon GE (2018). Treatment initiation for new episodes of depression in primary care settings. J Gen Intern Med.

[51] Fernández D, Vigo D, Sampson NA, Hwang I, Aguilar-Gaxiola S, Al-Hamzawi AO, Alonso J, Andrade LH, Bromet EJ, de Girolamo G, de Jonge P, Florescu S, Gureje O, Hinkov H, Hu C, Karam EG, Karam G, Kawakami N, Kiejna A, Kovess-Masfety V, Medina-Mora ME, Navarro-Mateu F, Ojagbemi A, O'Neill S, Piazza M, Posada-Villa J, Rapsey C, Williams DR, Xavier M, Ziv Y, Kessler RC, Haro JM (2021). Patterns of care and dropout rates from outpatient mental healthcare in low-, middle- and high-income countries from the World Health Organization's world mental health survey initiative. Psychol Med.

[52] Mathiasen K, Andersen TE, Lichtenstein MB, Ehlers LH, Riper H, Kleiboer A, Roessler KK (2022). The clinical effectiveness of blended cognitive behavioral therapy compared with face-to-face cognitive behavioral therapy for adult depression: randomized controlled noninferiority trial. J Med Internet Res.

[53] Munder T, Wilmers F, Leonhart R, Linster HW, Barth J (2010). Working Alliance Inventory-Short Revised (WAI-SR): psychometric properties in outpatients and inpatients. Clin Psychol Psychother.

[54] Henson P, Peck P, Torous J (2019). Considering the Therapeutic Alliance in Digital Mental Health Interventions. Harv Rev Psychiatry.

[55] Hatcher RL, Gillaspy JA (2006). Development and validation of a revised short version of the working alliance inventory. Psychother Res.

[56] Horvath AO, Greenberg LS (1989). Development and validation of the working alliance inventory. J Couns Psychol.

[57] Goldberg Simon B, Baldwin Scott A, Riordan Kevin M, Torous John, Dahl Cortland J, Davidson Richard J, Hirshberg Matthew J (2022). Alliance With an Unguided Smartphone App: Validation of the Digital Working Alliance Inventory. Assessment.

[58] Jasper K, Weise C, Conrad I, Andersson G, Hiller W, Kleinstäuber M (2014). The working alliance in a randomized controlled trial comparing Internet-based self-help and face-to-face cognitive behavior therapy for chronic tinnitus. Internet Interv.

[59] Kroenke K, Spitzer RL, Williams JB (2001). The PHQ-9: validity of a brief depression severity measure. J Gen Intern Med.

[60] Spitzer RL, Kroenke K, Williams JB, Löwe B (2006). A brief measure for assessing generalized anxiety disorder: the GAD-7. Arch Intern Med.

[61] Kroenke K, Wu J, Yu Z, Bair MJ, Kean J, Stump T, Monahan P (2016). Patient health questionnaire anxiety and depression scale: initial validation in three clinical trials. Psychosom Med.

[62] Macrynikola N, Chang S, Torous J (2024). Emotion regulation self-efficacy as a mechanism of alliance and outcomes in a brief, transdiagnostic digital mental health intervention: L'auto-efficacité de la régulation des émotions en tant que mécanisme d'alliance et de résultats dans une brève intervention transdiagnostique numérique en santé mentale. Can J Psychiatry.

[63] Gruber-Baldini AL, Velozo C, Romero S, Shulman LM (2017). Validation of the PROMIS® measures of self-efficacy for managing chronic conditions. Qual Life Res.

[64] Diener E, Wirtz D, Tov W, Kim-Prieto C, Choi DW, Oishi S, Biswas-Diener R (2009). New well-being measures: short scales to assess flourishing and positive and negative feelings. Soc Indic Res.

[65] Schotanus-Dijkstra M, Ten Klooster PM, Drossaert CH, Pieterse ME, Bolier L, Walburg JA, Bohlmeijer ET (2016). Validation of the flourishing scale in a sample of people with suboptimal levels of mental well-being. BMC Psychol.

[66] Luciano JV, Bertsch J, Salvador-Carulla L, Tomás JM, Fernández A, Pinto-Meza A, Haro JM, Palao DJ, Serrano-Blanco A (2010). Factor structure, internal consistency and construct validity of the Sheehan Disability Scale in a Spanish primary care sample. J Eval Clin Pract.

[67] Leon AC, Davis LL, Kraemer HC (2011). The role and interpretation of pilot studies in clinical research. J Psychiatr Res.

[68] Manea L, Gilbody S, McMillan D (2012). Optimal cut-off score for diagnosing depression with the Patient Health Questionnaire (PHQ-9): a meta-analysis. CMAJ.

[69] Kroenke Kurt, Spitzer Robert L, Williams Janet B W, Monahan Patrick O, Löwe Bernd (2007). Anxiety disorders in primary care: prevalence, impairment, comorbidity, and detection. Ann Intern Med.

[70] Johnson SU, Ulvenes PG, Øktedalen T, Hoffart A (2019). Psychometric properties of the General Anxiety Disorder 7-Item (GAD-7) scale in a heterogeneous psychiatric sample. Front Psychol.

[71] Connolly SL, Kuhn E, Possemato K, Torous J (2021). Digital clinics and mobile technology implementation for mental health care. Curr Psychiatry Rep.

[72] Fernandez E, Woldgabreal Y, Day A, Pham T, Gleich B, Aboujaoude E (2021). Live psychotherapy by video versus in-person: a meta-analysis of efficacy and its relationship to types and targets of treatment. Clin Psychol Psychother.

[73] Torous John, Lipschitz Jessica, Ng Michelle, Firth Joseph (2020). Dropout rates in clinical trials of smartphone apps for depressive symptoms: A systematic review and meta-analysis. J Affect Disord.

[74] Springer KS, Levy HC, Tolin DF (2018). Remission in CBT for adult anxiety disorders: a meta-analysis. Clin Psychol Rev.

[75] Cuijpers P, Karyotaki E, Ciharova M, Miguel C, Noma H, Furukawa TA (2021). The effects of psychotherapies for depression on response, remission, reliable change, and deterioration: a meta-analysis. Acta Psychiatr Scand.

[76] Kyanko KA, A Curry L, E Keene D, Sutherland R, Naik K, Busch SH (2022). Does primary care fill the gap in access to specialty mental health care? A mixed methods study. J Gen Intern Med.

[77] Abbott JH (2014). The distinction between randomized clinical trials (RCTs) and preliminary feasibility and pilot studies: what they are and are not. J Orthop Sports Phys Ther.

[78] Lungu A, Wickham RE, Chen S, Jun JJ, Leykin Y, Chen CE (2022). Component analysis of a synchronous and asynchronous blended care CBT intervention for symptoms of depression and anxiety: pragmatic retrospective study. Internet Interv.

[79] Chang Sarah, Alon Noy, Torous John (2023). An exploratory analysis of the effect size of the mobile mental health Application, mindLAMP. Digit Health.

[80] Wolitzky-Taylor K, LeBeau R, Arnaudova I, Barnes-Horowitz N, Gong-Guy E, Fears S, Congdon E, Freimer N, Craske M (2023). A novel and integrated digitally supported system of care for depression and anxiety: findings from an open trial. JMIR Ment Health.

